# Now Available Online: Final 2013–14 Influenza Vaccination Coverage Estimates for Selected Local Areas, States, and the United States

**Published:** 2014-09-19

Final 2013–14 influenza season vaccination coverage estimates are now available online at FluVaxView (http://www.cdc.gov/flu/fluvaxview). The online information includes estimates of the cumulative percentage of persons vaccinated by the end of each month, from July 2013 through May 2014, for select local areas, each state, each U.S. Department of Health and Human Services region, and the United States overall.

Analyses were conducted using National Immunization Survey influenza vaccination data for children aged 6 months–17 years and Behavioral Risk Factor Surveillance System data for adults aged ≥18 years. Estimates are provided by age group and race/ethnicity. These estimates are presented in an interactive report (http://www.cdc.gov/flu/fluvaxview/interactive.htm ) and complemented by an online summary report (http://www.cdc.gov/flu/fluvaxview/coverage-1314estimates.htm).

